# Supplementary material for: Association between oral microbiome and five types of respiratory infections: a two-sample Mendelian randomization study in east Asian population
Source: Front Microbiol. 2024 Apr 10;15:1392473. doi: 10.3389/fmicb.2024.1392473 (PMC11039966; doi:10.3389/fmicb.2024.1392473)
Supplement: Supplementary file 1 [file Data_Sheet_1.zip › Supplementary Materials/MR plots of saliva/Pneumonia/g__unclassified_mgs_2172/funnel.pdf]

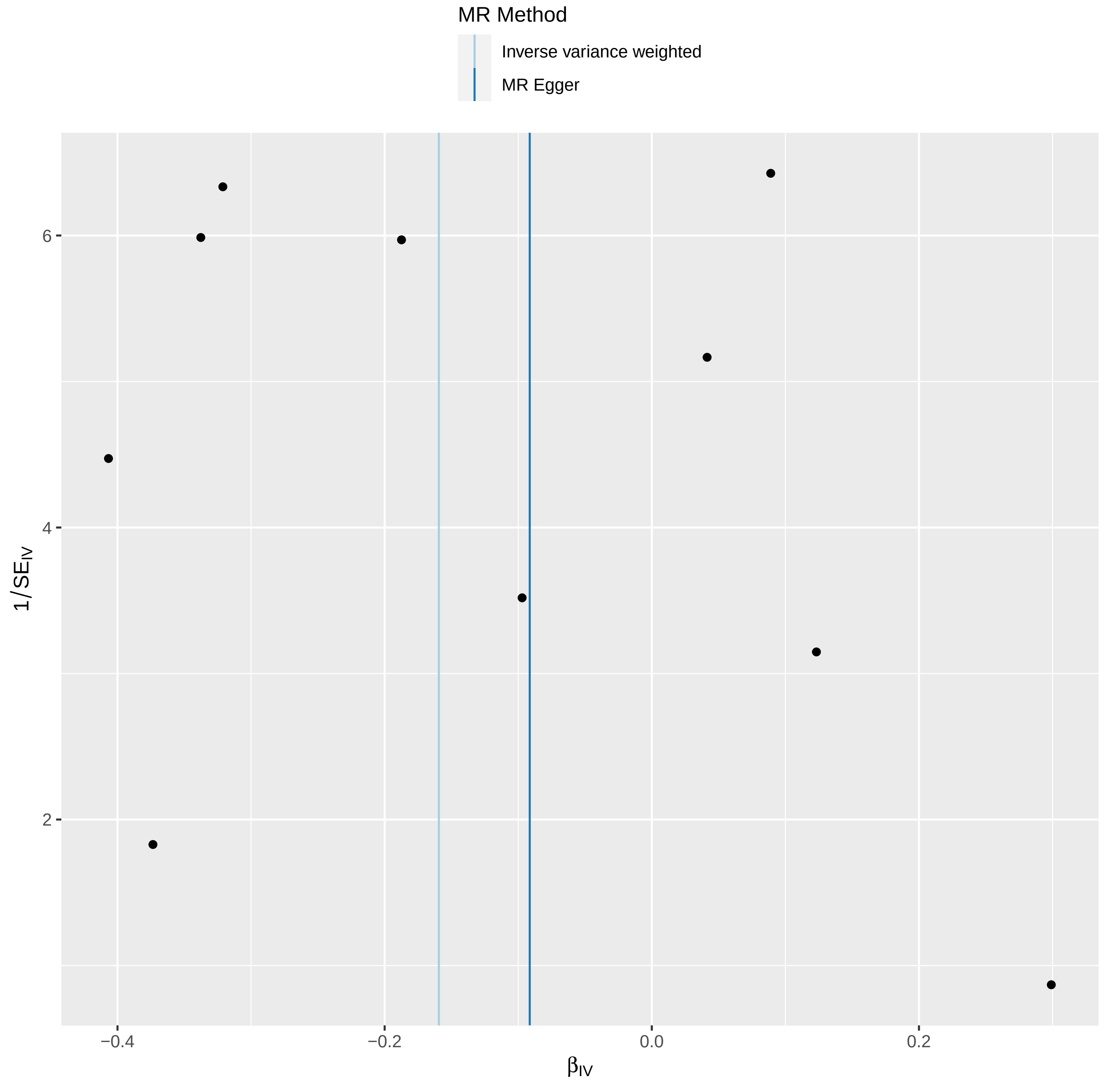

MR Method
Inverse variance weighted
MR Egger
6
4
2
1/SE_IV
−0.4
−0.2
0.0
0.2
β_IV
